# Gardnerella vaginalis in Symptomatic Men: Prevalence, Load, and Co-infections

**DOI:** 10.7759/cureus.94875

**Published:** 2025-10-18

**Authors:** Ali Egemen Avci, Hakan Cakir

**Affiliations:** 1 Department of Urology, Üsküdar University, Istanbul, TUR; 2 Department of Urology, Acıbadem Fulya Hospital, Istanbul, TUR

**Keywords:** co-infections, gardnerella vaginalis, multiplex pcr, sexually transmitted infections, urethritis

## Abstract

Introduction

*Gardnerella vaginalis*, traditionally linked to bacterial vaginosis (BV) in women, is increasingly detected in men with urethral symptoms. Its clinical relevance and co-infection patterns remain poorly defined. This study assessed the prevalence, quantitative load, and co-infections of *G. vaginalis*, and other urogenital pathogens in symptomatic men.

Methods

We retrospectively analyzed 418 symptomatic male patients using multiplex polymerase chain reaction (PCR), targeting 21 pathogens. Pathogen loads were reported as qualitative or quantitative (<10⁴ or ≥10⁴ copies/mL).

Results

At least one pathogen was detected in 239 (57.2%) patients (95% CI: 52.4-61.9). *G. vaginalis* was most frequently identified: 90 (21.5%), followed by *Chlamydia trachomatis*: 63 (15.1%), *Mycoplasma genitalium*: 45 (10.8%), and *Ureaplasma urealyticum*: 38 (9.1%). Quantitative analysis showed 105 patients with *G. vaginalis* <10⁴ copies/mL and 90 with ≥10⁴ copies/mL. Polymicrobial infections occurred in 81 (19.5%) patients, most often involving *G. vaginalis* with *U. urealyticum* (n = 13) or *U. parvum* (n = 11).

Conclusions

*G. vaginalis* was the leading pathogen in symptomatic men, frequently present at clinically significant loads and in co-infections. These findings support its inclusion in routine male sexually transmitted infection (STI) diagnostic panels.

## Introduction

Sexually transmitted infections (STIs) remain a major global public health problem, with an estimated 374 million new cases of curable STIs occurring annually worldwide [1]. While *Neisseria gonorrhoeae* and *Chlamydia trachomatis* are the most frequently identified etiologic agents of male urethritis, advances in molecular diagnostics have revealed the role of non-classical and polymicrobial pathogens in urogenital infections [2,3].

*Gardnerella vaginalis*, a facultative anaerobic Gram-variable bacterium, is a well-established cause of bacterial vaginosis (BV) in women [4]. Historically regarded as a component of the normal vaginal microbiota, *G. vaginalis* has been increasingly detected in the male urogenital tract, particularly among men presenting with urethral symptoms [5]. Reported prevalence rates in male populations vary widely - from less than 1% to over 25% - depending on patient selection, sampling site, and diagnostic methodology [6,7].

The development of multiplex polymerase chain reaction (PCR) assays has improved the detection of *G. vaginalis* and other fastidious organisms, which are often missed by traditional culture or Gram staining [8,9]. These assays also allow for quantitative assessment of pathogen load, which may help distinguish between transient colonization and clinically significant infection.

Emerging evidence suggests that *G. vaginalis* may be involved in polymicrobial infections in men, frequently co-occurring with *Ureaplasma* spp. and *Mycoplasma* spp., potentially through synergistic interactions that enhance pathogenicity [10,11]. In addition, male carriage of *G. vaginalis* has been implicated in the recurrence of BV among female partners, underscoring its potential role in sexual transmission cycles [12,13].

Despite increasing recognition, the clinical significance of *G. vaginalis* in men remains poorly defined, and data on its prevalence, quantitative load, and co-infection patterns in symptomatic male populations are limited.

The aim of this study was to determine the prevalence and quantitative load of *G. vaginalis* and other urogenital pathogens in symptomatic men using multiplex PCR and to evaluate the frequency and patterns of co-infections in this population.

## Materials and methods

Study design and population

This retrospective, descriptive study was conducted at the urology outpatient clinic of our institution between September 2022 and April 2025. The ethics committee approval for the study was obtained from the Memorial Hospital Local Ethics Committee on August 13, 2025, under number 2025/19. A total of 418 male patients presenting with symptoms suggestive of STIs were included. Only the first test result per patient was analyzed to avoid duplication.

Inclusion and exclusion criteria

Inclusion criteria were male patients aged ≥18 years and the presence of at least one of the following urogenital symptoms: urethral discharge, dysuria, penile pruritus, or penile discomfort. Exclusion criteria were asymptomatic individuals screened for other purposes; patients with incomplete clinical or laboratory records; duplicate samples from the same patient; patients who had used systemic or topical antibiotics, antifungals, or antiseptics within the last four weeks; patients with recent urethral catheterization, cystoscopy, or any other urological procedure within the previous three months; and detection of commensal flora organisms such as *Lactobacillus* spp., *Staphylococcus* spp., and *Enterobacteriaceae* spp., which were excluded from analysis.

Sample collection

Urethral swab samples were collected by trained healthcare professionals using sterile cotton-tipped applicators. The swab was inserted approximately 2-4 cm into the urethral meatus, rotated gently for several seconds, and then withdrawn. Samples were immediately placed into transport medium and sent to the microbiology laboratory for analysis. All samples were evaluated in the same laboratory in our hospital. In all patients, urethral Gram-stained smear microscopy was performed prior to PCR testing as part of routine clinical evaluation, although these results were not included in the current analysis. All samples were analyzed using a commercial multiplex real-time PCR system (Anyplex™ II STI-21; Seegene Inc., Seoul, Korea), performed on the CFX96™ Real-Time PCR Detection System (Bio-Rad Laboratories, Hercules, CA, USA).

Multiplex PCR analysis

All samples were tested using a commercial multiplex PCR assay capable of detecting 21 pathogens. The panel included *G. vaginalis*, *C. trachomatis*, *N. gonorrhoeae*, *Ureaplasma urealyticum*, *Mycoplasma genitalium*, *Mycoplasma hominis*, *Trichomonas vaginalis*, *Candida* spp., and Herpes simplex virus types 1 and 2 (HSV-1 and HSV-2).

Pathogen load was reported as negative, positive (qualitative only), quantitative <10⁴ copies/mL, or quantitative ≥10⁴ copies/mL. Commensal flora organisms were excluded from the final statistical analysis.

Statistical analysis

Data were entered into Microsoft Excel (Microsoft® Corp., Redmond, WA, USA) and analyzed using IBM SPSS Statistics for Windows, Version 21 (Released 2012; IBM Corp., Armonk, NY, USA). Categorical variables were expressed as frequencies and percentages, with 95% confidence intervals (CIs).

## Results

Overall pathogen detection

A total of 418 symptomatic male patients were tested using multiplex PCR. At least one urogenital pathogen was detected in 239 patients, corresponding to an overall positivity rate of 57.2% (95% CI: 52.4-61.9). The remaining 179 (42.8%) patients had no detectable pathogens above the reporting threshold.

Pathogen distribution and quantitative loads

The most frequently identified organism was *G. vaginalis*, detected in 90 (21.5%) patients, followed by *C. trachomatis* in 63 (15.1%) patients, *M. genitalium* in 45 (10.8%) patients, and *U. urealyticum* in 38 (9.1%) patients. Less common pathogens included *N. gonorrhoeae* (n = 19; 4.5%), *M. hominis* (n = 16; 3.8%), HSV type II (n = 11; 2.6%), *Candida* spp. (n = 2; 0.5%), and *T. vaginalis* (n = 2; 0.5%).

Quantitative analysis revealed that *G. vaginalis* DNA was detected at a load of <10⁴ copies/mL in 105 patients, and ≥10⁴ copies/mL in 90 patients. For *U. urealyticum*, 27 patients had <10⁴ copies/mL, and 38 patients had ≥10⁴ copies/mL, while for *U. parvum*, 25 and 31 patients were in these respective categories. Detailed pathogen-specific quantitative data are also summarized in Table 1, categorized as negative, qualitative positive, quantitative <10⁴, or quantitative ≥10⁴.

**Table 1:** Distribution of multiplex PCR results for each pathogen PCR, polymerase chain reaction

Pathogen	Negative, n(%)	Positive (qualitative), n(%)	Quantitative <10⁴, n(%)	Quantitative ≥10⁴, n(%)	Total positive, n(%)	95% CI
*Candida* spp.	446 (90.3%)	0 (0%)	46 (9.3%)	2 (0.4%)	48 (9.7%)	7.4-12.6
Chlamydia trachomatis	427 (86.3%)	68 (13.7%)	0 (0%)	0 (0%)	68 (13.7%)	11.0-17.1
Gardnerella vaginalis	299 (60.7%)	0 (0%)	105 (21.1%)	90 (18.2%)	195 (39.3%)	35.3-43.8
Herpes simplex virus (HSV) II	484 (97.8%)	11 (2.2%)	0 (0%)	0 (0%)	11 (2.2%)	1.2-3.9
Mycoplasma genitalium	446 (90.1%)	49 (9.9%)	0 (0%)	0 (0%)	49 (9.9%)	7.6-12.8
Mycoplasma hominis	468 (94.5%)	0 (0%)	11 (2.2%)	16 (3.3%)	27 (5.5%)	3.8-7.8
Neisseria gonorrhoeae	474 (95.8%)	21 (4.2%)	0 (0%)	0 (0%)	21 (4.2%)	2.8-6.4
Trichomonas vaginalis	493 (99.6%)	2 (0.4%)	0 (0%)	0 (0%)	2 (0.4%)	0.1-1.5
Ureaplasma urealyticum	429 (86.8%)	0 (0%)	27 (5.5%)	38 (7.7%)	65 (13.2%)	10.5-16.4

Single and multiple infections

Of the 239 patients with at least one pathogen detected, 134 (56.1%) patients had single infections, while 105 (43.9%) patients had multiple pathogens detected. Overall, polymicrobial infections were identified in 81 patients (19.5% of the total cohort, 33.9% of positive cases). The most common co-infection patterns were *G. vaginalis* + *U. urealyticum* (n = 13, 16.0% of co-infected cases) and *C. trachomatis* + *M. genitalium* (n = 6, 7.4%). Table 2 lists the three most frequent co-infection patterns.

**Table 2:** Most common co-infection patterns among positive cases

Co-infection pattern	Number of patients, n(%)	95% CI
*Gardnerella vaginalis* + *Ureaplasma urealyticum*	13 (16.0%)	9.6-25.5
*Chlamydia trachomatis* + *Mycoplasma genitalium*	6 (7.4%)	3.4-15.2

## Discussion

In this large cohort of symptomatic men evaluated by multiplex PCR, *G. vaginalis* emerged as the most frequently detected urogenital pathogen, with a prevalence of 21.5% [2,6]. This detection rate is higher than those reported in several earlier studies, where prevalence in male urethral samples ranged from 4% to 15% [2,6]. Khosropour et al. [2] reported an 11% prevalence in symptomatic men, while Swidsinski et al. [5] observed an increase in *G. vaginalis* detection from 5% with culture to 18% with PCR, underscoring the improved sensitivity of molecular methods. The use of urethral swab sampling in our study, coupled with a broad multiplex PCR panel, likely contributed to the higher detection rate observed.

Notably, *G. vaginalis* was present as the sole pathogen in 7.4% of all cases and in combination with at least one other pathogen in 60% of *G. vaginalis*-positive cases. The most frequent co-infecting organisms were *U. urealyticum* and *U. parvum*, consistent with prior findings suggesting potential microbial synergy between *G. vaginalis* and other fastidious organisms [10,11]. Sarier et al. [4] demonstrated that *G. vaginalis* and *M. hominis* display synergistic pathogenicity in BV, which may have parallels in male urogenital infections.

The quantitative load analysis revealed that a substantial proportion of *G. vaginalis* and *U. urealyticum* detections were at high organism counts (≥10⁴ copies/mL). High bacterial loads have been associated with symptomatic disease and an increased likelihood of transmission [12,13]. The 2019 randomized controlled trial by Vodstrcil et al., published in the New England Journal of Medicine, demonstrated that treatment of male partners significantly reduced recurrence rates of BV in women, highlighting the potential role of men as reservoirs for reinfection [3]. In our cohort, *G. vaginalis* detections with bacterial loads below 10⁴ copies/mL were considered low-level colonization rather than definitive infection, consistent with previous reports emphasizing that clinically significant urethral infection is more likely when the microbial load exceeds this threshold.

While *G. vaginalis* is frequently detected, its pathogenic role in men is not fully defined. Some studies suggest transient colonization without overt symptoms [14,15], whereas case reports document its association with urethritis and prostatitis, particularly in immunocompromised patients [6,7]. The high prevalence and significant co-infection rate observed in our cohort support considering *G. vaginalis* as a relevant pathogen in symptomatic men, especially when detected at high quantitative loads.

Empirical treatment regimens for *G. vaginalis* typically include metronidazole or clindamycin [16,17]. However, reports of metronidazole resistance in male isolates raise concerns about the efficacy of standard protocols [5,18]. Where feasible, targeted therapy guided by susceptibility testing may improve treatment outcomes, particularly in recurrent or treatment-resistant cases [19].

Limitations

This study has several limitations. Its retrospective design precluded correlation of microbiological findings with detailed clinical outcomes or partner infection status. Antimicrobial susceptibility testing was not performed, and follow-up data on treatment response were unavailable. Additionally, the study population was limited to symptomatic men presenting to a single tertiary center, which may limit generalizability. The study included only symptomatic men; therefore, asymptomatic carriers and the potential reservoir role of *G. vaginalis* in the general male population were not evaluated. The lack of antimicrobial susceptibility testing and follow-up data limited our ability to assess response to treatment or recurrence. Another limitation is that cases with *G. vaginalis* loads <10⁴ copies/mL may represent colonization rather than true infection, which could have led to overestimation of its prevalence in symptomatic men.

Implications for practice and research

Our findings reinforce the need to expand diagnostic panels for male STIs to include *G. vaginalis*, particularly in cases of persistent or recurrent urethritis with negative results for classical pathogens. The frequent detection of high bacterial loads and co-infections supports the potential value of combination antimicrobial therapy. Future prospective studies should explore the longitudinal persistence of *G. vaginalis* in men, its role in male-to-female transmission, and optimal management strategies. Routine inclusion of *G. vaginalis* in diagnostic panels may be particularly useful for patients with persistent urethritis unresponsive to empirical therapy.

## Conclusions

In this large cohort of symptomatic men, *G. vaginalis* was the most frequently detected urogenital pathogen, often present at high quantitative loads and in association with other sexually transmitted organisms. These findings suggest that *G. vaginalis* should be considered a relevant pathogen in male STI diagnostics, particularly when detected in high concentrations or in combination with other fastidious organisms.

Routine inclusion of *G. vaginalis* in multiplex PCR panels for men with urethral symptoms may improve diagnostic yield and guide more effective treatment strategies, including combination therapy in cases of polymicrobial infection. Further prospective studies are warranted to clarify its pathogenic role, persistence in the male urogenital tract, antimicrobial susceptibility patterns, and potential contribution to BV recurrence in female partners.

## References

[1] Tuddenham S, Hamill MM, Ghanem KG (2022). Diagnosis and treatment of sexually transmitted infections: a review. JAMA.

[2] Khosropour CM, Soge OO, Golden MR, Hughes JP, Barbee LA (2022). Incidence and duration of pharyngeal chlamydia among a cohort of men who have sex with men. Clin Infect Dis.

[3] Vodstrcil LA, Plummer EL, Fairley CK (2019). Male-partner treatment to prevent recurrence of bacterial vaginosis. N Engl J Med.

[4] Sarier M, Demir M, Turgut H, Hizel A, Emek M, Kukul E, Sepin N (2020). New approach to microscopy of gram-stained urethral smear: the kissing slide method. Sex Transm Dis.

[5] Swidsinski S, Moll WM, Swidsinski A (2022). Bacterial vaginosis-vaginal polymicrobial biofilms and dysbiosis. Dtsch Arztebl Int.

[6] Heide-Jørgensen U, Adelborg K, Kahlert J, Sørensen HT, Pedersen L (2018). Sampling strategies for selecting general population comparison cohorts. Clin Epidemiol.

[7] Turovskiy Y, Sutyak Noll K, Chikindas ML (2011). The aetiology of bacterial vaginosis. J Appl Microbiol.

[8] Wang X, Zhang Y, Liu T, Song C, Xue X, Liu J, Zhao H (2024). Development of a multiplex real-time quantitative PCR assay for detecting vaginal microbiota in Chinese women - China, 2021-2022. China CDC Wkly.

[9] Redelinghuys MJ, Geldenhuys J, Jung H, Kock MM (2020). Bacterial vaginosis: current diagnostic avenues and future opportunities. Front Cell Infect Microbiol.

[10] Rak K, Kiecka A, Białecka J, Kawalec A, Krzyściak P, Białecka A (2022). Retrospective analysis of the Ureaplasma spp. prevalence with reference to other genital tract infections in women of reproductive age. Pol J Microbiol.

[11] Nava-Memije K, Hernández-Cortez C, Ruiz-González V, Saldaña-Juárez CA, Medina-Islas Y, Dueñas-Domínguez RA, Aguilera-Arreola MG (2021). Bacterial vaginosis and sexually transmitted infections in an HIV-positive cohort. Front Reprod Health.

[12] Soni J, Sinha S, Pandey R (2024). Understanding bacterial pathogenicity: a closer look at the journey of harmful microbes. Front Microbiol.

[13] Stein RA, Bianchini EC (2022). Bacterial-viral interactions: a factor that facilitates transmission heterogeneities. FEMS Microbes.

[14] Vodstrcil LA, Muzny CA, Plummer EL, Sobel JD, Bradshaw CS (2021). Bacterial vaginosis: drivers of recurrence and challenges and opportunities in partner treatment. BMC Med.

[15] Vodstrcil LA, Plummer EL, Doyle M (2020). Treating male partners of women with bacterial vaginosis (StepUp): a protocol for a randomised controlled trial to assess the clinical effectiveness of male partner treatment for reducing the risk of BV recurrence. BMC Infect Dis.

[16] Huang SH, Hsu HC, Lee TF (2023). Prevalence, associated factors, and appropriateness of empirical treatment of trichomoniasis, bacterial vaginosis, and vulvovaginal candidiasis among women with vaginitis. Microbiol Spectr.

[17] Tse J, Chen J, Shi L, Cheng MM, Lillis R, Near AM (2025). Prevalence and accuracy of empiric treatment among patients with vaginitis symptoms in the United States. Sex Transm Dis.

[18] Alauzet C, Lozniewski A, Marchandin H (2019). Metronidazole resistance and nim genes in anaerobes: a review. Anaerobe.

[19] Tasse J, Dieppois G, Peyrane F, Tesse N (2021). Improving the ability of antimicrobial susceptibility tests to predict clinical outcome accurately: adding metabolic evasion to the equation. Drug Discov Today.

